# Comparison of the Transcriptional Profiles of Melanocytes from Dark and Light Skinned Individuals under Basal Conditions and Following Ultraviolet-B Irradiation

**DOI:** 10.1371/journal.pone.0134911

**Published:** 2015-08-05

**Authors:** Saioa López, Isabel Smith-Zubiaga, Alicia García de Galdeano, María Dolores Boyano, Oscar García, Jesús Gardeazábal, Conrado Martinez-Cadenas, Neskuts Izagirre, Concepción de la Rúa, Santos Alonso

**Affiliations:** 1 Department of Genetics, Physical Anthropology and Animal Physiology. University of the Basque Country UPV/EHU, Leioa, Bizkaia, Spain; 2 Department of Zoology and Animal Cell Biology, University of the Basque Country UPV/EHU, Leioa, Bizkaia, Spain; 3 Department of Cell Biology and Histology. University of the Basque Country UPV/EHU, Leioa, Bizkaia, Spain; 4 BioCruces Health Research Institute, Cruces University Hospital, Cruces-Barakaldo, Bizkaia, Spain; 5 Forensic Genetics Laboratory, Forensic Science Unit, Ertaintza-Basque Country Police, Erandio, Bizkaia, Spain; 6 Dermatology Service, BioCruces Health Research Institute, Cruces University Hospital, Cruces-Barakaldo, Bizkaia, Spain; 7 Department of Medicine, Jaume I University of Castellón, Castellón, Spain; German Cancer Research Center, GERMANY

## Abstract

We analysed the whole-genome transcriptional profile of 6 cell lines of dark melanocytes (DM) and 6 of light melanocytes (LM) at basal conditions and after ultraviolet-B (UVB) radiation at different time points to investigate the mechanisms by which melanocytes protect human skin from the damaging effects of UVB. Further, we assessed the effect of different keratinocyte-conditioned media (KCM+ and KCM-) on melanocytes. Our results suggest that an interaction between ribosomal proteins and the P53 signaling pathway may occur in response to UVB in both DM and LM. We also observed that DM and LM show differentially expressed genes after irradiation, in particular at the first 6h after UVB. These are mainly associated with inflammatory reactions, cell survival or melanoma. Furthermore, the culture with KCM+ compared with KCM- had a noticeable effect on LM. This effect includes the activation of various signaling pathways such as the mTOR pathway, involved in the regulation of cell metabolism, growth, proliferation and survival. Finally, the comparison of the transcriptional profiles between LM and DM under basal conditions, and the application of natural selection tests in human populations allowed us to support the significant evolutionary role of *MIF* and *ATP6V0B* in the pigmentary phenotype.

## Introduction

Melanocytes are melanin-producing cells that, in addition to hold a major role in the pigmentary phenotype, also play an important part in the protection of the skin against the damaging effects of ultraviolet-B (UVB) radiation, such as erythema, sunburn, development of malignant melanoma or other skin cancers [1–4].

The advent of cDNA microarray technology has allowed a preliminary understanding of the gene interactions and regulatory networks that take place in pigmentary cells in response to UVB [5–7]. One of the first reports using cDNA microarrays in various cell lines of human melanocytes for around 9,000 human genes [5] showed that various genes, mainly related to DNA/RNA synthesis and modification, ribosomal proteins or solute carriers and ionic channels, were modulated 4 hours after a single dose of UVB irradiation (100mJ/cm^2^). Later, Yang et al. [6] using a higher density microarray (with probes for approximately 47,000 transcripts), although for a single cell line of melanocytes, analysed the response of melanocytes to UVB. In contrast to Valéry et al. [5], Yang et al. [6] selected a 24-hour time point after UVB irradiation and reported a set of p53-target genes as major agents involved in the UV response.

However, many questions remain unsolved yet. For example, although the damage and the collateral consequences of UVB in the human skin are known to differ among individuals of different geographical origin and skin color [8], the different transcriptional responses that could arise between cultured human melanocytes from dark and light donors (hereinafter DM and LM, respectively) have not been completely elucidated. A recent work [9] performed a genome-wide transcriptome analysis of both DM and LM under basal conditions using RNA-Seq technology and found only 16 genes differentially expressed in the two cell types. However, their results could be somehow limited by the small number of melanocyte lines of each type (2 DM and 2 LM) analysed.

Furthermore, the response of melanocytes to UV radiation is known to be mediated by paracrine factors released by keratinocytes, which modulate the growth rate and dendricity of melanocytes, and which ultimately lead to an increased production of melanin [10–18]. In some cases this has been shown by growing melanocytes with keratinocyte conditioned media (KCM) *in vitro;* however, the procedure by which this medium is obtained varies among studies. Thus, while in some experiments this medium is collected after the irradiation of keratinocytes (hereinafter KCM+) [19–20], in other studies the medium is collected from keratinocytes that have not been previously irradiated (hereinafter KCM-) [10, 21–22]. Given the current lack of a consensus to define how to collect this media, we aimed to analyse the putative different responses that might arise when culturing melanocytes with either KCM- or KCM+.

Therefore, the objective of this work was to achieve a full view of the regulatory mechanisms that melanocytes undergo in response to UVB. Thus, we analyse herein the whole-genome transcriptional profile of dark and light melanocytes under basal conditions and after UVB irradiation at different time points (6, 12 and 24 hours) by means of gene expression microarrays. Further, we also aimed to assess the effect of different keratinocyte-conditioned media on melanocytes at a whole-genome level. With that aim, melanocytes were cultured in medium supplemented with keratinocyte-conditioned medium obtained both from non-irradiated (KCM-) and irradiated keratinocytes (KCM+).

This work outperforms previous studies in many regards: 1) we interrogate a large number of probes in the genome, including genes (28,000) and other non-coding RNAs (7,419), 2) we include both DM and LM and assess their transcriptional differences, 3) importantly, we use a relatively high number of biological replicates (6 cell lines of DM and 6 of LM), which minimises the noise from variability among individuals, 4) we perform a time-series analysis that detects both early and later stress responses and 5) we cultivate melanocytes with KCM- and KCM+ and assess their distinct influence.

## Materials and Methods

### Cell cultures

Human epidermal keratinocytes were purchased from Cascade Biologics (Life technologies, Carlsbad, CA, USA). Cells were cultured in EpiLife Medium supplemented with human keratinocyte growth supplement (HKGS). Human epidermal melanocytes were also purchased from Cascade Biologics: six lines isolated from lightly pigmented neonatal foreskin (LM), and six lines from darkly pigmented neonatal foreskin (DM). These melanocytes were cultivated in Medium 254 supplemented with 1% human melanocyte growth supplement (HMGS). All the cell lines were maintained in an incubator under an atmosphere of 5% CO_2_ at 37°C. Media were refreshed every two days.

### UV irradiation and Keratinocyte-conditioned medium

**Fig 1:**
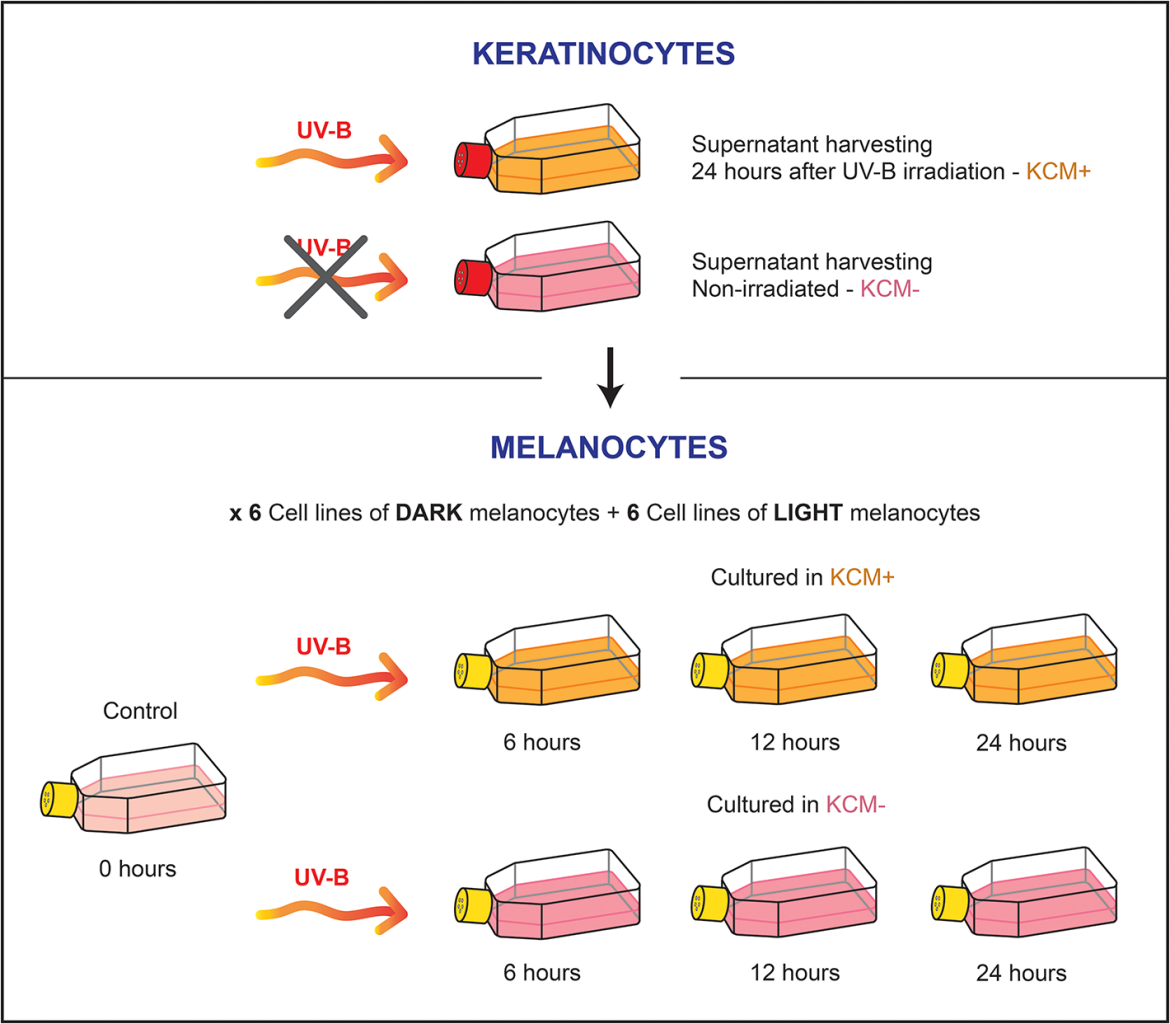
Graphical scheme of the experimental design.

UV irradiation was performed in an ICH2 photoreactor (LuzChem, Canada) at 37°C. Cultures were irradiated at 75 mJ/cm^2^ UVB, based on our previous work [20], as we observed that this dosage led to a notable physiological effect but did not affect cell viability in both keratinocytes and melanocytes. Keratinocyte supernatants were harvested from both non-irradiated (KCM-) and irradiated keratinocytes (24 hours after treatment) (KCM+) and kept frozen at -80°C until subsequent use. Subconfluent melanocyte cultures were cultivated in Medium 254 supplemented with HMGS and KCM+ or KCM- medium in a proportion 1:1. The following day they were irradiated with 75 mJ/cm^2^ of UVB, and harvested at 6, 12 and 24 hours post irradiation. We used non-irradiated control cultures that were covered by aluminium foil during irradiation (Fig 1).

### Microarrays

RNA from irradiated and non-irradiated melanocytes was extracted using the RNA extraction kit from Ambion (Life technologies). Samples were quantified using a UV/VIS NanoDrop 8000 (Thermo Fisher, Waltham, MA, USA), and RNA integrity was analysed through an Agilent 2100 Bioanalyzer using Agilent RNA 6000 Nano Chips (Agilent Technologies, Santa Clara, CA, USA). For each labeling reaction 100 ng RNA were used, with the Low input Quick Amp Labeling kit, one color (Agilent Technologies). First, total RNA was retrotranscribed using AffinityScript Reverse Transcriptase (Agilent Technologies) and Oligo dT primers linked to promoter T7. The synthesized double stranded cDNA was *in vitro* transcribed by T7 RNA polymerase with Cy3-CTP in order to achieve labeled and amplified cRNA. These samples were purified with RNeasy Mini kit columns (Qiagen, Hilden, Germany) and quantified to determinate the yield (which should be higher than 0.825 μg per reaction) and the specific activity of the fluorochrome Cyanine 3 (which should be higher than 6 pmol/μg). All the samples satisfied these requirements. Samples were analysed using SurePrint G3 Human GE Microarrays (Agilent Technologies), which have probes for 27,958 annotated genes and 7,419 long intergenic non-coding RNAs (lincRNAs). The hybridization step was performed using the SureHyb hybridization chamber (Agilent Technologies) and 600 ng of labeled cRNA samples, for 17 hours at 65°C and 10,000 rpms in a hybridization oven. Microarrays were stabilized with ozone-barrier slide covers (Agilent Technologies).

Image processing of the microarrays was performed by using the Agilent Feature Extraction software v10.7.3.1. This software performs 9 evaluation parameters to check the quality of the microarrays. The quality control parameters included, among others, the coefficient of variation of the processed signal from non-control probes and spike-ins (%CV), the percentage of outlier probes as regards the replicated probes population, the intensity of the signal of the negative controls and the limit of detection and linearity of the Spike-Ins signal.

### Microarray data pre-processing and normalization

Raw data were processed with GeneSpring GX software v11.5.1 (Agilent Technologies). Feature extraction flags were transformed as follows: if feature was not positive and significant, not uniform, not well above background or was a population outlier: compromised; if feature was saturated: not detected.

We performed a variance-stabilizing transformation of the data, which is a key step, but often not considered, in the pre-processing of microarrays data. Most of the subsequent statistical analyses assume that the data follow a normal distribution, with a constant variance independent of the mean of the data. Gene-expression microarray data, however, often have a variance that changes non-linearly with the mean, and thus, log transformations, which are used in the transformation of these data, can inflate the variance of observations near the background. Thus, our data were subjected to a DDHF (Data-Driven Haar-Fisz) transformation for variance stabilization with the R package DDHFm [23]. This method stabilizes the variance of replicated intensities from microarray data and produces transformed intensities that are much closer to the Gaussian distribution than other methods. Furthermore, it can be adapted to different or uncertain distributions, and therefore, it is ideal for the variance stabilization of microarray data.

Data were transformed to log base 2 and normalized following the quantile method [24]. Flag spot information in data files was used to filter probe sets. Entities in which more than 50% of samples in 1 out of any 7 conditions (0h, 6h KCM-, 12h KCM-, 24h KCM, 6h KCM+, 12h KCM+ and 24h KCM+) had “detected” flags were maintained for the analyses.

### Quality (QC) Metrics and Principal Component Analysis (PCA)

QC-Metrics was performed with GeneSpring GX software. Gene expression of the transformed and normalized data were subjected to unsupervised classification by means of Principal Component Analysis (PCA) as a preliminary exploratory approach to detect outliers, or the existence of defined clusters based on time points, pigmentation of the cells or the type of KCM used for culture. We used The Unscrambler X v10.3 (CAMO A/S, Trondheim, Norway) and applied the full cross validation method to estimate the stability and performance of the model.

### Comparison of expression profiles

Statistical analysis for the comparison of expression profiles was performed with SAM (Significance Analysis of Microarrays, [25]), using two class non-pairwise comparisons and 500 permutations in each test. The significance of the tests was given by the lowest False Discovery Rate at which the gene is called significant based on [26], adjusted for multiple tests.

### Pathway enrichment analysis

Enrichment analysis was performed using Web-based Gene Set Analysis Toolkit (WebGestalt) (http://bioinfo.vanderbilt.edu/-webgestalt/option.php), using all the probes analyzed in the microarray as the reference list, and The Kyoto Encyclopedia of Genes and Genomes (KEGG) database of pathways. The significance analysis was performed using the Hypergeometric test. P-values were corrected for multiple tests following the Bonferroni procedure. The minimum number of genes for enrichment was set at 5, and the significance level at Bonferroni adjusted-p<0.01, in order to be conservative, avoid false positives and achieve more robust results.

### Validation by RT-qPCR

We selected 6 genes showing a change in expression between dark and light melanocytes or after UV irradiation in the microarrays for validation with Real-Time quantitative PCR (RT-qPCR). cDNA was synthesized from 2 μg of total extracted RNA using the First Strand cDNA Synthesis Kit (ThermoFisher) and was used as a template for RT-qPCR analyses. Four different cell lines were analysed (2 of dark melanocytes, and 2 of light melanocytes). RT-qPCR reactions were performed with SYBR Green in a StepOne thermocycler (Life Technologies). Primer sequences (5'-3') were the following: MIFf_GAAGGCCATGAGCTGGTCT, MIFr_GGTTCCTCTCCGAGCTCAC, FDXRf_CTGAGGCAGAGTCGAGTGAAG, FDXRr_CCCGAAGCTCCTTAATGGTGA, TP53I3f_AATGCTTTCACGGAGCAAATTC, TP53I3r_TTCGGTCACTGGGTAGATTCT, ATP6VOBf_CCATCGGAACTACCATGCAGG, ATP6VOBr_TCCACAGAAGAGGTTAGACAGG, MDM2f_GAATCATCGGACTCAGGTACATC, MDM2r_TCTGTCTCACTAATTGCTCTCCT, RPL6f_ATTCCCGATCTGCCATGTATTC and RPL6r_TACCGCCGTTCTTGTCACC. Thermocycling conditions were optimized for each pair of primers to obtain 95–100% efficiency and r^2^>0.99 in the reaction. Gene expression was normalized to the housekeeping gene *GAPDH*. Each reaction was performed in triplicate and values were averaged to calculate relative expression levels.

### Selection tests

Preliminary screenings to detect deviations from neutrality were performed using the 1000 Genomes Selection Browser (http://hsb.upf.edu/) [27], which implements several neutrality tests (Tajima’s D, Fay & Wu’s H, Fu’s F, Fu and Li’s F*, Fu and Li’s D* and EHH, among many others) and provides genome based rank “p-values”, that help to identify which SNPs or regions have significantly high scores compared to the rest of the genome.

Further selection tests in candidate loci were performed with DnaSP [28]. We obtained the genotypes of the European (n = 760 chromosomes), African (n = 492) and Asian (n = 572) populations from the 1000 Genomes Project (Phase I May 2011) using SPSmart v5.1.1 (dbSNP build 132) [29]. The orthologous sequence of the chimpanzee was obtained from the UCSC Genome Browser and aligned to the human sequences with ClustalW. For each population, we calculated Tajima’s D, Fu & Li’s D and Fay & Wu’s H with DnaSP [28]. P-values for these tests were obtained using the interface for standard coalescent simulations conditioned on the number of segregating sites.

## Results and Discussion

### Quality metrics and PCA

QC-Metrics revealed 2 outlier arrays that did not satisfy the quality parameters: L_5.6K- (LM; replicate_5; 6h; KCM-) and L_4.24K- (LM; replicate_4; 24h; KCM-). Thus, those samples were removed from the subsequent statistical analyses.

**Fig 2:**
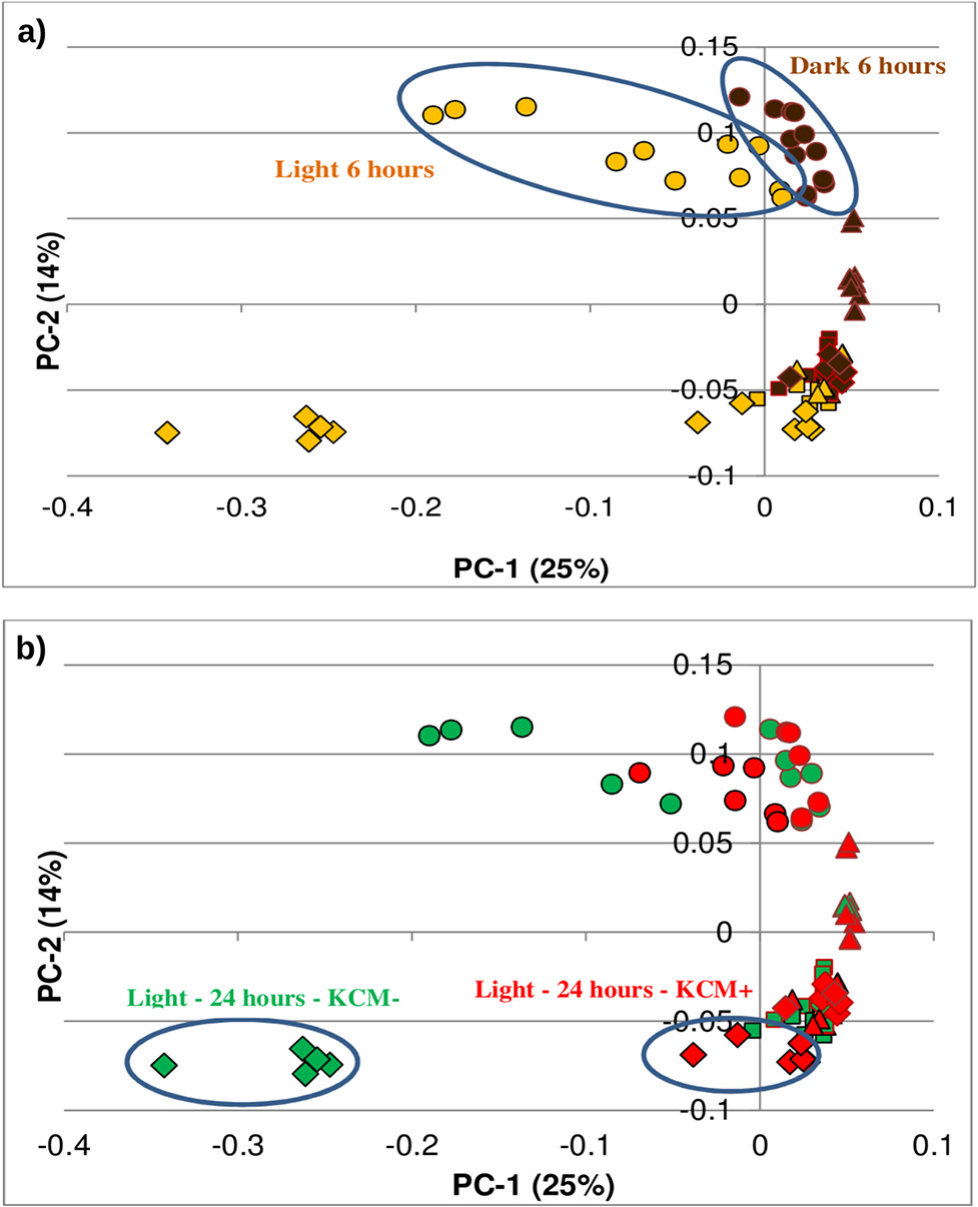
Principal Component Analysis. Charts a) and b) show the same 2-dimesional representation of the data according to the first 2 principal components, but colored according to different variables. Thus, in a) the effects of time (Squares: Time = 0; Dots: Time = 6 hours; Triangles: Time = 12 hours; Diamonds: Time = 24 hours) and pigmentation (Yellow = Light melanocytes; Brown = Dark melanocytes) are highlighted, while in b), it is the time (Squares: Time = 0; Dots: Time = 6 hours; Triangles: Time = 12 hours; Diamonds: Time = 24 hours) and the type of KCM used which are highlighted (Green: KCM-; Red: KCM+).

Second, we performed a Principal Component Analysis (PCA), an exploratory multivariate statistical technique for simplifying complex data sets [30], that has been used for the analysis of microarray data in search of outlier genes [31] or to identify temporal patterns in time-series analyses [32]. The PCA (Fig 2) allowed us to have an overview of the temporal patterns or differentially expressed genes between dark and light melanocytes, or between the culture with KCM+ or KCM-. It showed an apparent general homogeneity, revealing no additional potential outliers and a coherent clustering of our samples according to different variables, which was valuable to discard the presence of outliers or experimental errors. The PCA showed a time-point clustering defined by the second component, revealing a major separation of the samples at 6 hours, while the samples corresponding to 12 and 24 hours clustered close to the controls (0 hours), thus suggesting an early response from melanocytes to UVB that returned again to basal levels after the first 6 hours. At 6 hours we also observed a differential response according to pigmentation defined by the first component. At 24 hours, an apparent clustering regarding the culture of light melanocytes with KCM- or KCM+ was also noticed.

### Identification of differentially expressed probes after UVB

A total of 26,493 probes were examined per microarray. By means of SAM [25] we identified the statistically significant differentially expressed genes. Because probes may correspond to both genes and non-coding RNAs, we explicitly indicated when they corresponded to non-coding RNA. We first looked for common genes differentially expressed in DM and LM across time after UVB irradiation. We focused on the top upregulated and downregulated genes at 6, 12 and 24h, and in order to provide robust results, we identified those genes that were significantly up- or downregulated at more than one point (Tables 1 and 2). The adjusted p-value for all these genes was <0.0001.

**Table 1:** Most significantly upregulated genes at more than one time point (non-coding RNAs are indicated with *) (Bonferroni-adjusted p-value <0.0001).

Time points	Gene symbol	Accession number	Description
**6, 12 and 24h**	*FDXR*	NM_004110	ferredoxin reductase, nuclear gene encoding mitochondrial protein
	*EPHA2*	NM_004431	EPH receptor A2
	*RPL6*	NM_001024662	ribosomal protein L6
	*VWCE*	NM_152718	von Willebrand factor C and EGF domains
	*UBD*	NM_006398	ubiquitin D
	*CXCL1*	NM_001511	chemokine (C-X-C motif) ligand 1 (melanoma growth stimulating activity, alpha)
	*MDM2*	NM_002392	Mdm2 p53 binding protein homolog (mouse), transcript variant MDM2
	*RPL41*	NM_001035267	ribosomal protein L41
	*TNFRSF10C*	NM_003841	tumor necrosis factor receptor superfamily, member 10c
	*DDB2*	NM_000107	damage-specific DNA binding protein 2, 48kDa
	*GRM2*	NM_000839	glutamate receptor, metabotropic 2
	*TP53I3*	NM_004881	tumor protein p53 inducible protein 3
	*ISCU*	NM_014301	iron-sulfur cluster scaffold homolog (E. coli)
**6 and 12h**	*GADD45A*	NM_001924	growth arrest and DNA-damage-inducible, alpha
	*PLK3*	NM_004073	polo-like kinase 3
	*BTG2*	NM_006763	BTG family, member 2
	*TRIAP1*	NM_016399	TP53 regulated inhibitor of apoptosis 1
	*PIDD*	NM_145886	p53-induced death domain protein
	*CDKN1A*	NM_078467	cyclin-dependent kinase inhibitor 1A (p21, Cip1)
	*TP53INP1*	NM_033285	tumor protein p53 inducible nuclear protein 1
	*SESN1*	THC2527965	Sestrin 1, partial (68%)
	*BAG1*	NM_004323	BCL2-associated athanogene
	*XPC*	NM_004628	xeroderma pigmentosum, complementation group C
	*lincRNA chrX:64042150–64093950		
**12 and 24h**	*RPS2*	NM_002952	ribosomal protein S2
	*RPL26*	THC2550570	ribosomal protein L26, partial (91%)
	*PLXNB2*	NM_012401	plexin B2
	*KRT17*	NM_000422	keratin 17
	*ACTA2*	NM_001613	actin, alpha 2, smooth muscle, aorta
	*SULF2*	NM_018837	sulfatase 2
	*PVRL4*	NM_030916	poliovirus receptor-related 4
	*CSRP2*	NM_001321	cysteine and glycine-rich protein 2
	*DRAM1*	NM_018370	DNA-damage regulated autophagy modulator 1
	*BBC3*	NM_014417	BCL2 binding component 3
	*LOC344887	NR_033752	NmrA-like family domain containing 1 pseudogene, non-coding RNA
	*RELB*	NM_006509	v-rel reticuloendotheliosis viral oncogene homolog B
	*LOC642335	AK098072	cDNA FLJ40753 fis, clone TRACH2001188.
	*KIAA1324*	NM_020775	KIAA1324
	*NOV*	NM_002514	nephroblastoma overexpressed gene
	*RPS27L*	NM_015920	ribosomal protein S27-like
	*PRODH*	NM_016335	proline dehydrogenase (oxidase) 1, nuclear gene encoding mitochondrial protein
**6 and 24h**	*GDF15*	NM_004864	growth differentiation factor 15
	*NFKBIA*	NM_020529	nuclear factor of kappa light polypeptide gene enhancer in B-cells inhibitor, alpha

**Table 2:** Most significantly downregulated genes at more than one time point (non-coding RNAs are indicated with *) (Bonferroni-adjusted p-value <0.0001).

Time points	Gene symbol	Accession number	Description
**6, 12 and 24h**	*LGALS3*	NM_002306	lectin, galactoside-binding, soluble, 3
	*PDSS2*	NM_020381	prenyl (decaprenyl) diphosphate synthase, subunit 2
	*MAGI3*	NM_152900	membrane associated guanylate kinase, WW and PDZ domain containing 3
	*PSD3*	NM_015310	pleckstrin and Sec7 domain containing 3
	*lincRNA chr10:114583921–114587485 forward strand		
**6 and 12h**	*SBF2*	NM_030962	SET binding factor 2
	*XRCC4*	NM_022550	X-ray repair complementing defective repair in Chinese hamster cells 4
	*VAV2*	NM_003371	vav 2 guanine nucleotide exchange factor
	*ROR1*	NM_005012	receptor tyrosine kinase-like orphan receptor 1
	*ERC1*	NM_178040	ELKS/RAB6-interacting/CAST family member 1
	*lincRNA chr17:67547498–67549996 forward strand		
**12 and 24h**	*HMG20B*	NM_006339	high mobility group 20B
	*TUBA1B*	NM_006082	tubulin, alpha 1b
	*SMYD3*	NM_022743	SET and MYND domain containing 3
	*SCFD2*	NM_152540	sec1 family domain containing 2
	*VTI1A*	NM_145206	vesicle transport through interaction with t-SNAREs homolog 1A (yeast)
	*PRR4*	NM_007244	proline rich 4 (lacrimal)
	*PARD3*	NM_019619	par-3 partitioning defective 3 homolog (C. elegans)
	*RABGAP1L*	NM_014857	RAB GTPase activating protein 1-like
	*TTC28*	NM_001145418	tetratricopeptide repeat domain 28
	*PCCA*	NM_000282	propionyl CoA carboxylase, alpha polypeptide
	*MAN1C1*	NM_020379	mannosidase, alpha, class 1C, member 1
	*A4GALT*	NM_017436	alpha 1,4-galactosyltransferase
	*MSRA*	NM_012331	methionine sulfoxide reductase A
	*ANO4*	NM_178826	anoctamin 4
	*SSBP2*	NM_012446	single-stranded DNA binding protein 2
	*STX8*	NM_004853	syntaxin 8
	*REXO1*	NM_020695	REX1, RNA exonuclease 1 homolog (S. cerevisiae)
	*SH3KBP1*	NM_031892	SH3-domain kinase binding protein 1
	*BBS9*	NM_198428	Bardet-Biedl syndrome 9
	*BCKDHB*	NM_000056	branched chain keto acid dehydrogenase E1, beta polypeptide
	*SORCS1*	NM_001206572	sortilin-related VPS10 domain containing receptor 1
	*TPK1*	NM_022445	thiamin pyrophosphokinase 1
	*LINGO2*	NM_152570	leucine rich repeat and Ig domain containing 2
	*FRY*	NM_023037	furry homolog (Drosophila)
	*PDE3B*	NM_000922	phosphodiesterase 3B, cGMP-inhibited
	*KCNQ2*	NM_172109	potassium voltage-gated channel, KQT-like subfamily, member 2
	*PPIA*	THC2525667	Peptidylprolyl isomerase A
	*lincRNA chr2:7214634–7218011 reverse strand		
	*lincRNA chr4:79892901–80229698 forward strand		
	*lincRNA chr18:42263052–42278652 forward strand		
	*lincRNA chr18:74178337–74203637 forward strand		
	*lincRNA chr7:125564239–125734564 forward strand		
**6 and 24h**	*FAM78B*	NM_001017961	family with sequence similarity 78, member B
	*VAV3*	NM_006113	vav 3 guanine nucleotide exchange factor

#### Common upregulated genes after UVB irradiation

Some of the genes included in this category (Table 1) have already been reported to be associated with the response to ultraviolet irradiation, which gives robustness to our inferences. The most significantly upregulated gene was *FDXR*, which serves as the first electron transfer protein in all the mitochondrial P450 systems, and has been reported to be upregulated in response to UV irradiation damage in dendritic cells [**33**] and melanocytes [**6**]. Importantly, we also observed several genes involved in the regulation of the cell cycle, in the response to stress, in the repair of DNA damage caused by UV that can lead to xeroderma pigmentosum, as well as genes that are associated with melanoma.

We also observed several genes that take part in the regulation of the cell cycle and in the cellular response to stress and that are directly or indirectly involved in the p53 pathway. Some of them modulate P53-mediated apoptosis or cell death in response to stresses like UV irradiation or DNA damage, like *TP53I3*, *PLK3*, *TRIAP1*, *PIDD*, *CDKN1A*, *TP53INP1*, *SESN1*, *BBC3*, *TNFRSF10C*, *DRAM1* and *MDM2*. Other genes that also are upregulated and participate in UV irradiation-induced apoptosis include *RELB* and *EPHA2*.

Another group of the upregulated genes are components of the nucleotide excision repair (NER) pathway that are associated with the reparation of DNA damage caused by UV, and which include *XPC* or *DDB2*. Malfunction of these genes can lead to xeroderma pigmentosum, a recessive disease that is characterized by an increased sensitivity to UV light and a high predisposition for skin cancer development. Several other genes among the top upregulated ones have been reported to be directly or indirectly associated with melanoma, such as *BTG2*, *BAG1*, *CXCL1*, *PLXNB2*, *CSRP2*, *PRODH* or *GDF15*. Various genes that encode ribosomal proteins such as *RPL6*, *RPL41*, *RPS2*, *RPL26* and *RPS27L* were also observed.

Intriguingly, we observed an upregulation of the gene *NOV*. The protein encoded by this gene is of particular relevance as it has been reported to be essential for the correct development and growth of melanocytes [34]. During development, melanocytes migrate to the epidermis and attach to the basement membrane upon contact with keratinocytes. Development of melanocytes must be tightly regulated and must remain at stable levels in relation to keratinocytes. Fukunaga-Kalabis et al. [34] discovered that *NOV* is upregulated in melanocytes upon contact with keratinocytes in culture, mediating the growth inhibition of melanocytes in order to regulate their spatial location and number. Our results suggest that this gene could also participate in the regulation of melanocytes' growth in response to UVB, most likely by inhibiting their proliferation and allowing either the triggering of cell death or reparation events.

#### Common downregulated genes after UVB irradiation

Among the top downregulated genes in response to UVB irradiation (Table 2), of particular interest was *LGALS3*, which plays a role in numerous cellular functions including apoptosis, innate immunity, cell adhesion and T-cell regulation, and regulates the expression of several genes that are aberrantly expressed in highly aggressive melanoma cells [**35**]. Another interesting downregulated gene was *PDSS2*, which encodes the prenyl side-chain of coenzyme Q (CoQ), one of the key elements in the respiratory chain. As it has been reported that UV light depletes CoQ10 from the skin [**36**], this consequently suggests that the downregulation of *PDSS2* could be one of the first inducers of reactive oxygen species (ROS) production and, consequently, of oxidative damage to the DNA in the cells ultimately caused by UVB.

Several neuron-related genes were also downregulated by UVB, like ROR1, ERC1, PARD3, SORCS1, LINGO2 and KCNQ2. As neurons and melanocytes share the same embryonic origin (the neural crest) they might likely share some cell regulatory processes [37]. Our results suggest that some genes that are involved in neuronal growth or migration could also be found in melanocytes exerting similar functions. In this regard, it was noticeable that a handful of lincRNAs were also downregulated after UVB irradiation. Although for most lincRNAs biological functions and mechanisms of action remain unknown, our results suggest that some lincRNAs are likely key elements of the regulatory machinery of melanocytes.

### Differential transcriptional profile of dark and light melanocytes 6 hours after UVB

As inferred from the unsupervised PCA (Fig 2) the greatest differences among melanocytes were found at 6 hours after UVB irradiation. From that point, melanocytes seem to have started to go back to the basal state. Thus, we focused on determining the differential expression between DM and LM at 6 hours after irradiation (the results for other time points can be found in S1–S4 Tables).

#### Upregulated genes in LM vs DM 6 hours after UVB irradiation

The significant upregulation of *EDA2R* in LM (Table 3) suggests a putative role for this gene in response to UVB irradiation in light skinned individuals. *EDA2R* has been reported to be affected by recent natural positive selection [**38**–**39**], and its paralog, *EDAR*, has been strongly associated with skin pigmentation variability in humans [**40**]. Strikingly, the intergenic region between *EDA2R* and the next gene on the same chromosome (*AR)* is the most divergent genomic segment between Africans and East Asians in the human genome [**41**].

**Table 3:** Top 50 upregulated genes in LM vs DM at 6 hours after UVB irradiation (non-coding RNAs are indicated with *) (Bonferroni-adjusted p-value <0.0001).

Gene symbol	Accession number	Description
*CDKN2A*	NM_000077	cyclin-dependent kinase inhibitor 2A (melanoma, p16, inhibits CDK4)
*SNAR-A3*	NR_024214	small ILF3/NF90-associated RNA A3, small nuclear RNA
*KIAA1377*	NM_020802	uncharacterized protein KIAA1377
*TTC18*	NM_145170	tetratricopeptide repeat domain 18
*NCAM1*	NM_001242607	neural cell adhesion molecule 1, tr. variant 5
*HOXB13*	NM_006361	homeobox B13
*PYCARD*	NM_013258	PYD and CARD domain containing
*CSRNP3*	NM_001172173	cysteine-serine-rich nuclear protein 3
*PKMYT1*	NM_182687	protein kinase, membrane associated tyrosine/threonine 1
*lincRNA:chr1:85932812–85974062 reverse strand		
*QPCT*	NM_012413	glutaminyl-peptide cyclotransferase
*EDA2R*	NM_001242310	ectodysplasin A2 receptor
*SGMS1*	NM_147156	sphingomyelin synthase 1
*RPL37A*	NM_000998	ribosomal protein L37a
*HIST1H4L*	NM_003546	histone cluster 1, H4l
*GDPD1*	NM_182569	glycerophosphodiester phosphodiesterase domain containing 1
*HIST1H3B*	NM_003537	histone cluster 1, H3b
*lincRNA:chr10:133738235–133744210 forward strand		
*GDPD5*	NM_030792	Glycerophosphodiester phosphodiesterase domain containing 5
*SUV420H2*	NM_032701	suppressor of variegation 4–20 homolog 2 (Drosophila)
*MOBKL2B*	NM_024761	MOB1, Mps One Binder kinase activator-like 2B (yeast)
*MXD3*	NM_031300	MAX dimerization protein 3
*TUBA1B*	NM_006082	tubulin, alpha 1b
*SAC3D1*	NM_013299	SAC3 domain containing 1
*LOC390595	NM_001163692	ubiquitin-associated protein 1-like
*SNORD15A*	NR_000005	small nucleolar RNA, C/D box 15A, small nucleolar RNA
*ZNF711*	NM_021998	zinc finger protein 711
*lincRNA:chrX:102139220–102156619 forward strand		
*LTBP3*	ENST00000525443	latent transforming growth factor beta binding protein 3
*FAM164A*	NM_016010	family with sequence similarity 164, member A
*ARHGEF10*	ENST00000523711	Rho guanine nucleotide exchange factor (GEF) 10
*S100B*	NM_006272	S100 calcium binding protein B
*HMGN2*	NM_005517	high mobility group nucleosomal binding domain 2
*C1orf15-NBL1*	NM_001204088	C1ORF15-NBL1 readthrough
*GALNT14*	NM_024572	polypeptide N-acetylgalactosaminyltransferase 14 (GalNAc-T14)
*SPTLC3*	NM_018327	serine palmitoyltransferase, long chain base subunit 3
*IFI27L2*	NM_032036	interferon, alpha-inducible protein 27-like 2
*RNF6*	NM_005977	ring finger protein (C3H2C3 type) 6
*TUBB8*	NM_177987	tubulin, beta 8
*PDGFRL*	NM_006207	platelet-derived growth factor receptor-like
*ARPC5*	ENST00000367534	actin related protein 2/3 complex, subunit 5, 16kDa
*PTGDS*	NM_000954	prostaglandin D2 synthase 21kDa (brain)
*SLC2A13*	NM_052885	solute carrier family 2 (facilitated glucose transporter), member 13
*CTSF*	NM_003793	Cathepsin F
*C1orf133	NR_024337	SERTAD4 antisense RNA 1
*WFDC1*	NM_021197	WAP four-disulfide core domain 1
*TUBG1*	NM_001070	tubulin, gamma 1
*SLC12A8*	NM_024628	solute carrier family 12, member 8
*CXADR*	NM_001338	coxsackie virus and adenovirus receptor
*SOX5*	NM_152989	SRY (sex determining region Y)-box 5

We also identified as upregulated some genes related to melanoma, like *CDKN2A*, whose expression has already been reported to be induced by UV radiation [42] and which could be conferring light skinned individuals a higher susceptibility to develop melanoma in response to UV radiation, as well as several neuron-related genes and genes associated with the formation of tubulin, the major constituent of microtubules of the cytoskeleton, and which have been shown to mediate the transport the melanosomes inside the cell [43].

#### Upregulated genes in DM vs LM 6 hours after UVB irradiation

Next, we focused on the most significant upregulated genes in DM vs LM (Table 4). In this case, we found several genes involved in inflammatory reactions. Some of them have been reported to be particularly involved in sunburn or inflammatory skin reactions in response to UVB, like IL6 [**44**], PTGS2 [**45**] or CCL2 [**46**]. Similarly to LM, DM also showed an upregulation of various genes involved in melanoma progression as well as several genes related to the development of the central nervous system and neuronal processes.

**Table 4:** Top 50 upregulated genes in DM vs LM at 6 hours after UVB irradiation (non-coding RNAs are indicated with *) (Bonferroni-adjusted p-value <0.0001).

Gene symbol	Accession numer	Description
*HUMRPL26X*	THC2550570	ribosomal protein L26 partial (91%)
*MMP1*	NM_002421	matrix metallopeptidase 1 (interstitial collagenase)
*RPL7A*	NM_000972	ribosomal protein L7a
*CCL2*	NM_002982	chemokine (C-C motif) ligand 2
*NPTX2*	NM_002523	neuronal pentraxin II
*COL6A2*	NM_058174	collagen, type VI, alpha 2
*S100A4*	NM_002961	S100 calcium binding protein A4
*CXCL1*	NM_001511	chemokine (C-X-C motif) ligand 1 (melanoma growth stimulating activity, alpha)
*IL6*	NM_000600	interleukin 6 (interferon, beta 2)
*TMEM158*	NM_015444	transmembrane protein 158 (gene/pseudogene)
*PAMR1*	NM_015430	peptidase domain containing associated with muscle regeneration 1
*TMEM8B*	NM_001042590	collagen, type VI, alpha 2
*CXCL5*	NM_002994	chemokine (C-X-C motif) ligand 5
*TDRD9*	NM_153046	tudor domain containing 9
*ANGPTL4*	NM_139314	angiopoietin-like 4
*PMEPA1*	NM_020182	prostate transmembrane protein, androgen induced 1
*MEG3	NR_003531	maternally expressed 3 (non-protein coding) non-coding RNA
*C1D	ENST00000412019	C1D nuclear receptor corepressor pseudogene
*C1QBP*	NM_001212	complement component 1, q subcomponent binding protein
*FAM27A	NR_024060	family with sequence similarity 27, member A, non-coding RNA
*TMEM132A*	NM_017870	transmembrane protein 132A
*SLC16A2*	NM_006517	solute carrier family 16, member 2 (monocarboxylic acid transporter 8)
*ANPEP*	NM_001150	alanyl (membrane) aminopeptidase
*lincRNA:chr2:239460050–239536125 forward strand		
*FN1*	NM_054034	fibronectin 1
*MYOF*	NM_133337	myoferlin
*NR4A3*	NM_173200	nuclear receptor subfamily 4, group A, member 3
*EFS*	NM_005864	embryonal Fyn-associated substrate
*GRTP1*	NM_024719	growth hormone regulated TBC protein 1
*TNFRSF11B*	NM_002546	tumor necrosis factor receptor superfamily, member 11b
*DEF6*	NM_022047	differentially expressed in FDCP 6 homolog (mouse)
*FAM27A	NR_024060	family with sequence similarity 27, member A, non-coding RNA
*PTGS2*	NM_000963	prostaglandin-endoperoxide synthase 2
*GUCA1B*	NM_002098	guanylate cyclase activator 1B (retina)
*IL1RAP*	NM_134470	interleukin 1 receptor accessory protein
*SUSD3*	NM_145006	sushi domain containing 3
*lincRNA:chr17:73585552–73590170 forward strand		
*TNNI3*	NM_000363	troponin I type 3 (cardiac)
*C10orf116*	NM_006829	chromosome 10 open reading frame 116
*SPON2*	NM_012445	spondin 2, extracellular matrix protein
*LYPD1*	NM_144586	LY6/PLAUR domain containing 1
*IL27RA*	NM_004843	interleukin 27 receptor, alpha
*FUT1*	NM_000148	fucosyltransferase 1 (galactoside 2-alpha-L-fucosyltransferase, H blood group)
*CARD9*	NM_052813	caspase recruitment domain family, member 9 1
*SLC22A17*	NM_016609	solute carrier family 22, member 17
*IL11*	NM_000641	interleukin 11
*TNFAIP2*	NM_006291	tumor necrosis factor, alpha-induced protein 2
*C15orf48*	NM_032413	chromosome 15 open reading frame 48
*NT5E*	NM_002526	5'-nucleotidase, ecto (CD73)
*CXCL3*	NM_002090	chemokine (C-X-C motif) ligand 3

An interesting observation was the upregulation of the *lincRNA MEG3*. The expression of this *lincRNA*, stimulated by cyclic-AMP (cAMP), seems to act as a growth suppressor in tumour cells through the activation of *P53* [47]. As UVR is one of the main stimulatory sources of cAMP, these results suggest that in response to UV radiation, DM upregulate the expression of *MEG3* via cAMP liberation, which could confer protection against melanoma.

#### Pathway enrichment analysis

Focusing on single loci allows deciphering the differentially expressed genes between different categories (i.e. time or pigmentation). However, although this is useful to determine which genes can be key in the response to UVB, the full biological mechanisms underlying this response may remain obscure. Therefore, we used WebGestalt to look for pathways in KEGG (Kyoto Encyclopedia of Genes and Genomes) that were differentially overrepresented at each time point (Table 5) in LM and DM. We observed that the most significant pathways overrepresented among the upregulated genes corresponded to *ribosome* and *P53 signaling pathway* in both LM and DM. Further analyses using other databases of pathways implemented in WebGestalt (Pathway Commons and Wikipathways) confirmed the involvement of these two pathways in the response to UVB (data not shown).

**Table 5:** KEGG pathway enrichment analysis of the upregulated and downregulated genes in DM and LM after UVB irradiation.

	DM		LM	
	Pathway	Adj-p	Pathway	Adj-p
**Upregulated at 6h**	p53 signaling pathway	9.49E-07	p53 signaling pathway	7.04E-07
	Ribosome	3.00E-04	Ribosome	2.43E-06
**Upregulated at 12h**	Ribosome	3.22E-11	Systemic lupus erythematosus	1.16E-06
	RNA transport	7.11E-07	Ribosome	1.47E-06
	Ribosome biogenesis in eukaryotes	7.00E-04	p53 signaling pathway	1.10E-03
	p53 signaling pathway	2.13E-02	Mismatch repair	5.40E-03
			Pathways in cancer	6.90E-03
			Apoptosis	7.90E-03
**Upregulated at 24h**	Ribosome	1.06E-09	Ribosome	7.38E-06
	p53 signaling pathway	1.50E-03		
**Downregulated at 6h**	Adherens junction	1.51E-08	Ubiquitin mediated proteolysis	6.84E-12
	Ubiquitin mediated proteolysis	7.21E-08	Adherens junction	5.23E-11
	Wnt signaling pathway	9.12E-06	Endocytosis	6.14E-06
	Colorectal cancer	3.53E-05	Wnt signaling pathway	3.14E-05
	Progesterone-mediated oocyte maturation	4.04E-05	Progesterone-mediated oocyte maturation	5.32E-05
	Endocytosis	6.24E-05	Cell cycle	7.36E-05
	Pathways in cancer	7.60E-05	Insulin signaling pathway	5.00E-04
	Systemic lupus erythematosus	1.00E-04	Oocyte meiosis	7.00E-04
	Cell cycle	9.00E-04	Pathways in cancer	7.00E-04
	Oocyte meiosis	1.00E-03	Colorectal cancer	1.20E-03
	Endometrial cancer	1.10E-03	Neurotrophin signaling pathway	2.60E-03
	Fc epsilon RI signaling pathway	1.70E-03	Fc gamma R-mediated phagocytosis	8.60E-03
	B cell receptor signaling pathway	1.80E-03	Endometrial cancer	1.18E-02
	ErbB signaling pathway	1.90E-03	Chronic myeloid leukemia	1.54E-02
	Fc gamma R-mediated phagocytosis	2.20E-03	Bacterial invasion of epithelial cells	1.76E-02
	T cell receptor signaling pathway	2.50E-03	Fc epsilon RI signaling pathway	2.11E-02
	Insulin signaling pathway	5.30E-03	Chemokine signaling pathway	4.22E-02
	Neurotrophin signaling pathway	1.97E-02	ErbB signaling pathway	4.22E-02
			T cell receptor signaling pathway	4.22E-02
**Downregulated at 12h**	Metabolic pathways	4.43E-05	Protein processing in endoplasmic reticulum	2.00E-03
	Adherens junction	1.00E-04		
	Fc gamma R-mediated phagocytosis	3.00E-04		
	Propanoate metabolism	1.10E-03		
	Protein processing in endoplasmic reticulum	2.70E-03		
	Systemic lupus erythematosus	3.10E-03		
	Purine metabolism	4.90E-03		
	Regulation of actin cytoskeleton	1.20E-02		
	Valine, leucine and isoleucine degradation	4.30E-02		
	Pyrimidine metabolism	4.30E-02		
**Downregulated at 24h**			Cell cycle	2.20E-08
			Systemic lupus erythematosus	8.56E-06
	-		DNA replication	2.54E-05
			Oocyte meiosis	2.00E-03
			Lysine degradation	2.12E-02

**Fig 3:**
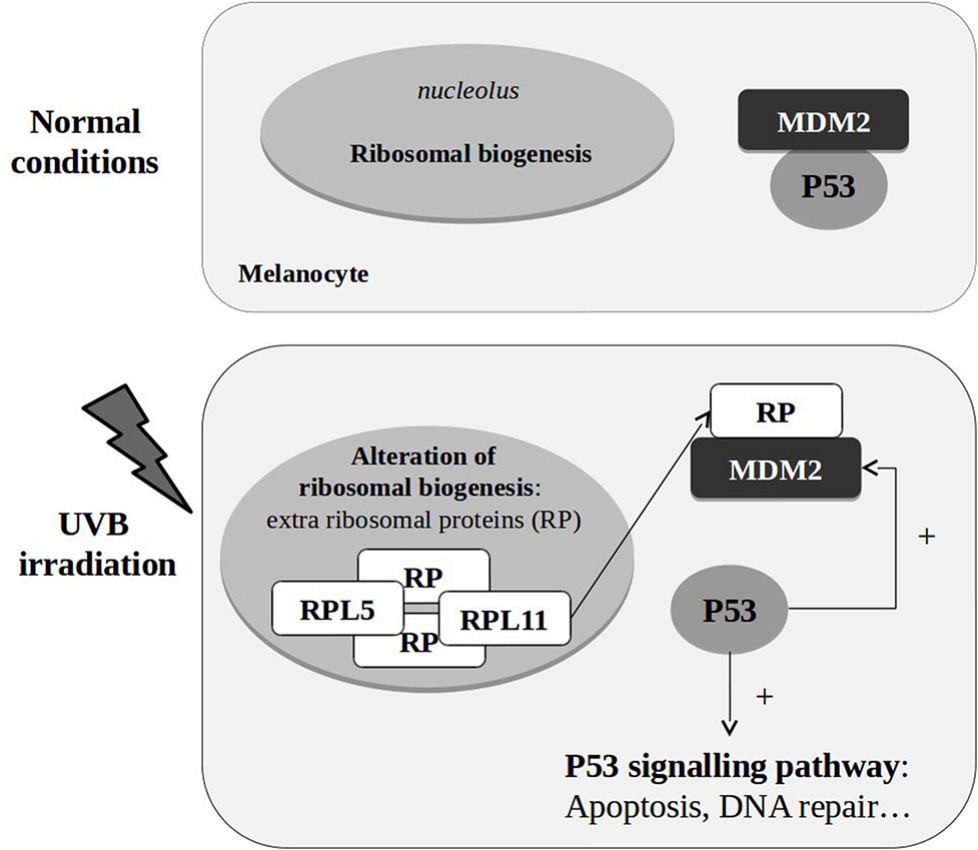
Proposed mechanism for the involvement of ribosomal proteins, MDM2 and p53 signaling pathway in the response to UVB irradiation.

The role of *P53*, a tumour suppressor that promotes either cell cycle arrest and DNA repair, apoptosis or senescence [48] in the response to UVB has already been reported [6]. Our results are consistent with the proposed mechanism of P53 pathway regulation by ribosomal proteins [49–51]. Thus, we propose that under stress, there is an upregulation of the ribosomal biogenesis leading to an excess of ribosomal proteins that do not participate in the assembly of ribosomes. Instead, these translocate to the nucleoplasm where they interact with MDM2. Under normal conditions, MDM2 binds to the tumour suppressor P53 inhibiting its transcription. But if ribosomal proteins bind to MDM2, then the inhibition of P53 exerted by MDM2 is suppressed. The upregulation of *MDM2* is usually modulated by P53 after the activation of P53-dependent targets, in order to inhibit the activity of P53 and thus restore the normal growth of the cell. However, if the stressing conditions are not completely restablished or DNA damage still exists in the cell, ribosomal proteins could continue interacting with MDM2 to allow to maintain the expression of *P53* (Fig 3). Among the ribosomal proteins that can bind to MDM2 are RPL5 [52] and RPL11 [53], both of which were among the upregulated ribosomal genes in this work.

On the other hand, several pathways were significantly overrepresented among the genes that were downregulated after UVB exposure, especially in the first 6h in both DM and LM (Table 5). Interestingly, the *adherens junction* pathway was downregulated in both cell types and at different time points. Adherens junctions play an important role maintaining skin homeostasis by mediating the interaction of melanocytes and keratinocytes, which control the proliferation of melanocytes [54], thus preventing the development and progression of melanoma [55].

### The effect of keratinocyte conditioned medium

Next, we assessed the expression profiles of melanocytes supplemented with keratinocyte-conditioned medium obtained both from non-irradiated (KCM-) and irradiated keratinocytes (KCM+). Again, differentially expressed genes were obtained with SAM and we performed a pathway enrichment analysis (Table 6). We did not observe any significantly downregulated pathways in melanocytes growing with KCM+ vs KCM-. As regards the upregulated pathways, various pathways were affected in LM, most of them related to *signaling pathways*. We did not detect any upregulated pathway in DM, which suggests that DMs could have lower requirements for keratinocyte-derived factors to start the response mechanisms against UV irradiation. On the contrary, LM show a significant upregulation of several pathways when cultivated with KCM+ compared to KCM-, which could suggest that for these cells, the type or the concentration of factors present in KCM- is not enough and they require more factors to engage in certain metabolic activities.

**Table 6:** KEGG pathway enrichment analysis for genes upregulated in the culture with KCM+ vs KCM- (significant pathways for the downregulated ones were not observed).

	LM		DM	
	Pathway	Adj-p	Pathway	Adj-p
**6h**	Ubiquitin mediated proteolysis	1.00E-03	-	
	mTOR signaling pathway	4.34E-02		
**12h**	Neurotrophin signaling pathway	8.03E-05	-	
	Phosphatidylinositol signaling system	5.50E-03		
	Endocytosis	2.28E-02		
**24h**	RNA transport	1.50E-03	Focal adhesion	4.85E-05
	Insulin signaling pathway	4.70E-03		
	Ubiquitin mediated proteolysis	4.90E-03		
	Homologous recombination	7.60E-03		
	mRNA surveillance pathway	8.30E-03		
	SNARE interactions in vesicular transport	9.50E-03		
	Spliceosome	1.24E-02		
	Cell cycle	1.66E-02		
	Pyrimidine metabolism	1.89E-02		
	Ribosome biogenesis in eukaryotes	2.23E-02		
	Wnt signaling pathway	4.46E-02		

Among the results obtained, of particular interest was the *mTOR signaling pathway*, which was upregulated in LM at 6h after UVB irradiation growing in KCM+. mTOR can be activated by UVR through the triggering of growth factor receptors bearing receptor tyrosine kinase (RTK) activity [56–58] like keratinocyte-derived EGF, FGF or HGF. mTOR signaling reciprocally interacts with p53 as a life/death regulator of irradiated skin cells. It has been shown that upon activation by UVR, mTOR can inhibit apoptosis and force cell cycle transition, or drive cells into senescence. This work reveals that the keratinocyte-derived factors activate the mTOR signaling pathway in LM to induce cell proliferation, consistent with the upregulation of cell cycle observed later at 24 hours. We propose that in this case mTOR forces cell cycle transition. This, however, could increase the susceptibility to develop melanoma, especially if DNA damage caused by UVB has not been repaired yet. In fact, mTOR pathway has been shown to be activated in the majority of malignant melanomas [59]. The fact that this pathway was activated in LM in culture with KCM+ suggests that some keratinocyte-derived factors, secreted after the irradiation of keratinocytes with UVB, could also be at the base of melanocytes’ malignancy.

Other signaling pathways that were upregulated in the presence of KCM+ are also activated by keratinocyte derived factors, such as the neurotrophin signaling pathway, which is activated by NGF and promotes the survival of melanocytes.

### Identification of differentially expressed genes in LM and DM under basal conditions

In order to identify putative candidate genes involved in normal pigmentation variability, we compared the transcriptional profiles of DM and LM under basal conditions (i.e. at time 0, without irradiation). No significantly overrepresented pathways were observed here. Therefore, we focused on the 50 most significant genes in each category (Tables 7 and 8). The most significant genes upregulated in LM (Table 7) were *ATP6V0B* and *ATP6VOD1*. These encode two components of the V-ATPase, which is responsible for maintaining an adequate acidic environment within melanosomes for the synthesis of melanin [60]. The most significantly upregulated gene in DM compared to LM (Table 8) was *MIF*. *MIF* has been identified as a regulator of melanogenesis, as it shows D-dopachrome tautomerase activity, which transforms D-dopachrome, dopaminechrome or its derivatives into precursors of melanin or neuromelanin [61]. It has also been suggested that *MIF* mediates melanogenesis in the skin through the activation of protease-activated receptor (PAR-2) and stem cell factor (SCF) expression in keratinocytes after exposure to UVB [62]. Interestingly, Polimanty et al [63] reported a correlation between the CNV 22q11.23 containing the gene *MIF* with environmental variables. In particular, they suggested that *MIF*-related gene dosage could be associated with the adaptation to UVR, and that darker skins were correlated with haplotypes carrying no deletions. Copy number variability, and the higher frequency of deletions at this locus in light skinned individuals could be leading to a decreased *MIF* gene dosage, as observed in this work.

**Table 7:** Top 50 upregulated genes in LM vs DM under basal conditions (non- coding RNAs are indicated with *) (bonferroni-adjusted p-value <0.0001).

Locus name	Accession number	Description
*ATP6V0B*	NM_004047	ATPase, H+ transporting, lysosomal 21kDa, V0 subunit b
*ATP6V0D1*	NM_004691	ATPase, H+ transporting, lysosomal 38kDa, V0 subunit d1
*FUT6*	NM_000150	fucosyltransferase 6 (alpha (1,3) fucosyltransferase)
*SLC16A12*	NM_213606	solute carrier family 16, member 12
*HSCB*	NM_172002	HscB iron-sulfur cluster co-chaperone homolog (E. coli)
*ZNF865*	NM_001195605	zinc finger protein 865
*EFS*	NM_005864	embryonal Fyn-associated substrate
*KRT31*	NM_002277	keratin 31
*RPL36A-HNRNPH2*	NM_001199973	RPL36A-HNRNPH2 readthrough
*JMJD5*	NM_001145348	jumonji domain containing 5
*HIST1H4C*	NM_003542	histone cluster 1, H4c
*GFRA3*	NM_001496	GDNF family receptor alpha 3
*KIAA1826*	NM_032424	KIAA1826
*DNAJC19*	NM_145261	DnaJ (Hsp40) homolog, subfamily C, member 19
*HAP1*	NM_177977	huntingtin-associated protein 1
*UXS1*	NM_025076	UDP-glucuronate decarboxylase 1
*SCAMP1*	NM_004866	secretory carrier membrane protein 1
*LTA4H*	NM_000895	leukotriene A4 hydrolase
*DYNC1LI1*	NM_016141	dynein, cytoplasmic 1, light intermediate chain 1
*LRRFIP2*	NM_006309	leucine rich repeat (in FLII) interacting protein 2
*C10orf88*	NM_024942	chromosome 10 open reading frame 88
*PDCD1*	NM_005018	programmed cell death 1
*MZT2A*	ENST00000491265	mitotic spindle organizing protein 2A
*MCM3*	NM_002388	minichromosome maintenance complex component 3
*C6orf163*	NM_001010868	chromosome 6 open reading frame 163
*DTX4*	NM_015177	deltex homolog 4 (Drosophila)
*CLCN6*	NM_021735	chloride channel 6 (CLCN6)
*RFK*	NM_018339	riboflavin kinase
*WHSC2*	NM_005663	Wolf-Hirschhorn syndrome candidate 2
*FGFRL1*	NM_001004356	fibroblast growth factor receptor-like 1
*BTBD6*	NM_033271	BTB (POZ) domain containing 6
*N4BP1*	NM_153029	NEDD4 binding protein 1
*MAP2K6*	NM_002758	mitogen-activated protein kinase kinase 6
*POMP*	NM_015932	proteasome maturation protein
*GABPA*	NM_002040	GA binding protein transcription factor, alpha subunit 60kDa
*UPF3A*	NM_023011	UPF3 regulator of nonsense transcripts homolog A (yeast)
*PLEKHA3*	NM_019091	pleckstrin homology domain containing, family A member 3
*CD276*	NM_001024736	CD276 molecule (CD276)
*ENTPD2*	NM_203468	ectonucleoside triphosphate diphosphohydrolase 2
*DEDD*	NM_032998	death effector domain containing
*FAM70B*	ENST00000375348	family with sequence similarity 70, member B
*MCM5*	NM_006739	minichromosome maintenance complex component 5
LOC100131257*	NR_034022	zinc finger protein 655 pseudogene
*SCARNA13*	NR_003002	small Cajal body-specific RNA 13
*SMNDC1*	NM_005871	survival motor neuron domain containing 1
*CALML4*	NM_033429	calmodulin-like 4
*C1orf131*	NM_152379	chromosome 1 open reading frame 131
*RNGTT*	NM_003800	RNA guanylyltransferase and 5'-phosphatase
*KCNQ3*	NM_004519	potassium voltage-gated channel, KQT-like subfamily, member 3
*WASF3*	NM_006646	WAS protein family, member 3

**Table 8:** Top 50 upregulated genes in DM vs LM under basal conditions (non- coding RNAs are indicated with *) (bonferroni-adjusted p-value <0.0001).

Locus name	Accession number	Description
*MIF*	NM_002415	macrophage migration inhibitory factor
TTC19*	ENST00000395886	tetratricopeptide repeat domain 19
*CYTB*	ENST00000361789	mitochondrially encoded cytochrome b
*NBEA*	NM_015678	neurobeachin
*CTSO*	NM_001334	cathepsin O
*SNORA23*	NR_002962	small nucleolar RNA, H/ACA box 23
*PMP22*	NM_000304	peripheral myelin protein 22
*CXCL1*	NM_001511	chemokine ligand (melanoma growth stimulating activity, alpha)
*CDKN2A*	NM_058197	cyclin-dependent kinase inhibitor 2A (melanoma, p16)
*LDB3*	NM_001171610	LIM domain binding 3
*MIPEP*	NM_005932	mitochondrial intermediate peptidase
*MAPK8*	NM_139047	mitogen-activated protein kinase 8
*SNORD15A*	NR_000005	small nucleolar RNA, C/D box 15A
SNAR-A3*	NR_024214	small ILF3/NF90-associated RNA A3
*ASCC1*	NM_015947	activating signal cointegrator 1 complex subunit 1
*ZNF235*	NM_004234	zinc finger protein 235
*MBIP*	NM_001144891	MAP3K12 binding inhibitory protein 1
*C13orf38*	NM_001198908	chromosome 13 open reading frame 38
LOC100132707*	NR_024477	hypothetical LOC100132707
*UTRN*	NM_007124	utrophin
*CALM2*	NM_001743	calmodulin 2 (phosphorylase kinase, delta)
MOCS1*	NM_005943	molybdenum cofactor synthesis 1
*ZNF212*	NM_012256	zinc finger protein 212
*KIAA0090*	NM_015047	KIAA0090 (KIAA0090)
*SNORD3B-1*	NR_003271	small nucleolar RNA, C/D box 3B-1
*HLX*	NM_021958	H2.0-like homeobox
*C9orf72*	NM_145005	chromosome 9 open reading frame 72
*SEC23B*	NM_032985	Sec23 homolog B (S. cerevisiae)
*WRAP73*	NM_017818	WD repeat containing, antisense to TP73
*MAN1A1*	NM_005907	mannosidase, alpha, class 1A, member 1
*S100B*	NM_006272	S100 calcium binding protein B
*CCDC93*	NM_019044	coiled-coil domain containing 93
*ZNF3*	NM_032924	zinc finger protein 3
*FTH1*	NM_002032	ferritin, heavy polypeptide 1
*RAB30*	NM_014488	RAB30, member RAS oncogene family
*RDM1*	NM_001034836	RAD52 motif 1
*BTAF1*	NM_003972	BTAF1 RNA polymerase II,
*HLA-F*	NM_018950	major histocompatibility complex, class I, F
*CABYR*	NM_012189	calcium binding tyrosine-(Y)-phosphorylation regulated
*MAP4K2*	NM_004579	mitogen-activated protein kinase 2
*PRPF18*	ENST00000320054	PRP18 pre-mRNA processing factor 18 homolog (S. cerevisiae)
*CALM3*	NM_005184	calmodulin 3 (phosphorylase kinase, delta)
*ALKBH4*	NM_017621	alkB, alkylation repair homolog 4 (E. coli)
LOC399744*	NR_024497	hypothetical LOC399744
*SETD6*	NM_024860	SET domain containing 6
*SH3TC2*	NM_024577	SH3 domain and tetratricopeptide repeats 2
*ARHGAP35*	NM_004491	Rho GTPase activating protein 35
*CHCHD6*	NM_032343	coiled-coil-helix-coiled-coil-helix domain containing 6
*RC3H2*	NM_018835	ring finger and CCCH-type domains 2
*WDR46*	NM_005452	WD repeat domain 46

For the other genes in Tables 7 and 8 we did not find any evident direct correlation with pigmentary phenotype.

### Validation by RT-qPCR

**Fig 4:**
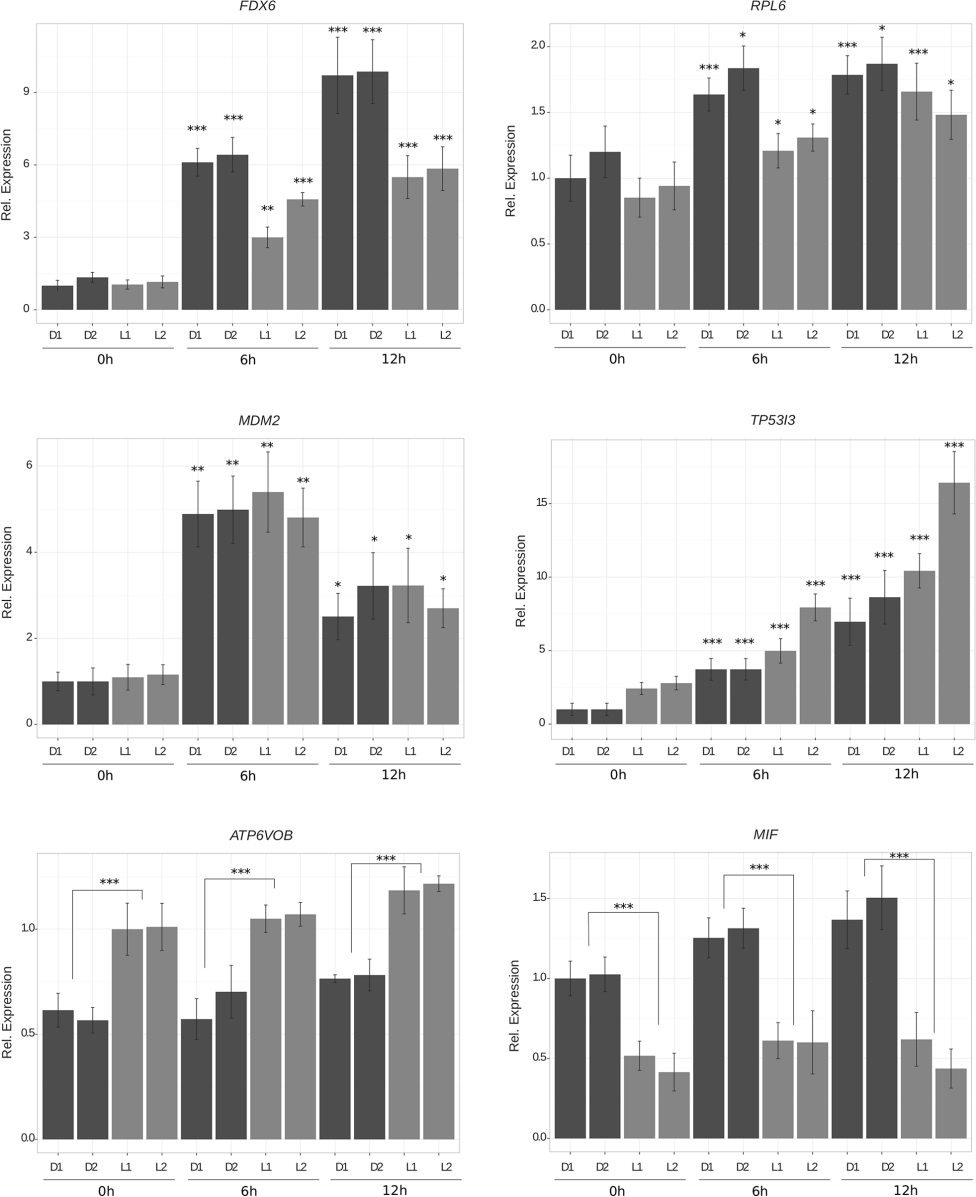
Gene expression of genes *FDX6*, *RPL6*, *MDM2*, *TP53I3*, *ATP6VOB* and *MIF* assessed by RT-qPCR. Unpaired t-test; *** p<0.0001; ** p<0.001; * p<0.01.

Six genes were selected for validation of the microarrays' results, which showed either a change of expression after UV treatment or a differential expression between LM and DM (*ATP6VOB*, *TP53I3*, *MDM2*, *MIF*, *RPL6* and *FDXR*), and measured their expression levels by quantitative real-time PCR (RT-qPCR). We assessed the expression of 4 melanocytic cell lines (2 DM and 2 LM) at basal conditions and at 6 and 12 hours after UVB irradiation. The expression patterns and direction of changes of all of the genes were consistent with the microarray data (Fig 4), observing a significant increase in the expression of *TP53I3*, *MDM2*, *RPL6* and *FDXR* in both LM and DM after UVB. The expression analysis of *ATP6VOB* and *MIF* also supported the differential expression of these genes by LM and DM, being *ATP6VOB* more expressed by LM, while *MIF* was more significantly expressed by DM (both at basal conditions and after UVB irradiation).

### Natural selection tests

In order to assess the biological relevance of the genes that were differentially expressed between DM and LM under basal conditions, we performed evolutionary neutrality tests on these genes (Tables 7 and 8) using the populations from the 1000 Genomes Project (1KGP). For this, we performed a first screening of different neutrality tests using the 1000 Genomes Selection Browser to identify putative signatures of selection. After multiple test correction, the gene *ATP6V0D1* (ATPase, H+ Transporting, Lysosomal 38kDa, V0 Subunit D1) seemed to deviate from neutrality in the European populations.

Further neutrality tests using DnaSP [28] supported significant signatures of selection acting on *ATP6V0D1* in Europeans (Tajima's D: -2.31, p-value = 0; Fay & Wu's H: -10.66, p-value = 0.001), thus suggesting that this gene might be involved in human pigmentary phenotype. This reinforces the notion that selective pressures can shape pigmentation variability by driving the evolution of melanosomal genes. So, besides the well-known *OCA2*, *SLC45A2* and *SLC24A5*, we support *ATP6V0D1* as an additional melanosomal-membrane gene that has been subjected to selective pressures and might be involved in pigmentation variability in Europeans.

No deviations from neutrality were detected in any population for the *MIF* gene (data not shown). However, we should take into account that *MIF* is embedded in a CNV [63] and in a previous work we observed how a variation in copy number can interfere with neutrality tests by altering the frequencies of polymorphisms leading to an excess of detected homozygosity [64]. A loss of copies would result in apparent homozygosity, and duplications of one allele would mask possible variant alleles in sequencing or genotyping experiments. Therefore, although with the available tools and our knowledge we cannot detect deviations from neutrality, we cannot still exclude the possibility that this gene is under selection.

## Conclusions

We have provided an overview of the most significant genes that are up and downregulated in response to UVB irradiation and revealed the interaction of ribosomal proteins and P53 signaling pathway in the response to UVB in both DM and LM. We have also observed that DM and LM show differentially expressed genes after irradiation and in particular in the first 6 hours. These are mainly associated with inflammatory skin reactions, cell survival or melanoma. Furthermore, the culture with KCM+ compared with KCM- had a noticeable effect on LM, but not in DM, triggering various signaling pathways in LM such as the mTOR signaling pathway. And importantly, the comparison of the transcriptional profile of LM and DM under basal conditions allowed us to highlight the significant involvement of *MIF* and *ATP6V0B* in the normal variability of human skin pigmentation.

## Supporting Information

S1 Table Top 50 upregulated genes in LM vs DM 12 hours after UVB. (PDF)

S2 Table Top 50 upregulated genes in DM vs LM 12 hours after UVB. (PDF)

S3 Table Top 50 upregulated genes in LM vs DM 24 hours after UVB. (PDF)

S4 Table Top 50 upregulated genes in DM vs LM 24 hours after UVB. (PDF)
