# Supplementary material for: Comparison of the Transcriptional Profiles of Melanocytes from Dark and Light Skinned Individuals under Basal Conditions and Following Ultraviolet-B Irradiation
Source: PLoS One. 2015 Aug 5;10(8):e0134911. doi: 10.1371/journal.pone.0134911 (PMC4526690; doi:10.1371/journal.pone.0134911)
Supplement: S1 Table — (PDF) [file pone.0134911.s001.pdf]

S1 Table. Top 50 upregulated genes in LM vs DM 12 hours after UVB (non- coding RNAs are indicated with *) (bonferroni-adjusted p-value <0.0001).

Locus name	Accession number	Description
RPLP2	NM_001004	*Homo sapiens* ribosomal protein, large, P2
ACTB	NM_001101	*Homo sapiens* actin, beta
RPS28	NM_001031	*Homo sapiens* ribosomal protein S28
FTH1	NM_002032	*Homo sapiens* ferritin, heavy polypeptide 1
STK39	NM_013233	*Homo sapiens* serine threonine kinase 39
FUT8	NM_178155	*Homo sapiens* fucosyltransferase 8 (alpha (1,6) fucosyltransferase)
ENOX2	NM_182314	*Homo sapiens* ecto-NOX disulfide-thiol exchanger 2
**LOC100128054*	NR_033969	
WDR7	NM_015285	*Homo sapiens* WD repeat domain 7
RNGTT	NM_003800	*Homo sapiens* RNA guanylyltransferase and 5'-phosphatase
LMO7	NM_005358	*Homo sapiens* LIM domain 7
CDKN3	NM_005192	*Homo sapiens* cyclin-dependent kinase inhibitor 3
SCLT1	NM_144643	*Homo sapiens* sodium channel and clathrin linker 1
PPM1H	NM_020700	*Homo sapiens* protein phosphatase, Mg2+/Mn2+ dependent, 1H
TMEM117	NM_032256	*Homo sapiens* transmembrane protein 117
TBC1D19	NM_018317	*Homo sapiens* TBC1 domain family, member 19
DNAJC1	NM_022365	*Homo sapiens* DnaJ (Hsp40) homolog, subfamily C, member 1
NEURL1B	NM_001142651	*Homo sapiens* neuralized homolog 1B (Drosophila)
CCNB1	NM_031966	*Homo sapiens* cyclin B1
CDKL2	NM_003948	*Homo sapiens* cyclin-dependent kinase-like 2 (CDC2-related kinase)
ATP6V0A4	NM_020632	*Homo sapiens* ATPase, H+ transporting, lysosomal V0 subunit a4
EFCAB11	NM_145231	*Homo sapiens* EF-hand calcium binding domain 11
CCDC91	NM_018318	*Homo sapiens* coiled-coil domain containing 91
CWC27	NM_005869	*Homo sapiens* CWC27 spliceosome-associated protein homolog (S. cerevisiae)
PKP4	NM_003628	*Homo sapiens* plakophilin 4
ANK3	NM_020987	*Homo sapiens* ankyrin 3, node of Ranvier (ankyrin G)
FTH1	NM_002032	*Homo sapiens* ferritin, heavy polypeptide 1
SCD5	NM_001037582	*Homo sapiens* stearoyl-CoA desaturase 5
ABAT	NM_000663	*Homo sapiens* 4-aminobutyrate aminotransferase
DEPDC1B	NM_018369	*Homo sapiens* DEP domain containing 1B
SLC24A1	NM_004727	*Homo sapiens* solute carrier family 24 sodium/potassium/calcium exchanger)
CADM3	NM_021189	*Homo sapiens* cell adhesion molecule 3
FIG4	NM_014845	*Homo sapiens* FIG4 homolog, SAC1 lipid phosphatase domain containing (S. cerevisiae)
EPB41L4A	NM_022140	*Homo sapiens* erythrocyte membrane protein band 4.1 like 4A
HMGN2	NM_005517	*Homo sapiens* high mobility group nucleosomal binding domain 2
EXOC6	NM_019053	*Homo sapiens* exocyst complex component 6
GHR	NM_000163	*Homo sapiens* growth hormone receptor
DZIP3	NM_014648	*Homo sapiens* DAZ interacting protein 3, zinc finger
MYO9A	NM_006901	*Homo sapiens* myosin IXA
COG5	NM_006348	*Homo sapiens* component of oligomeric golgi complex 5
HMGN2	NM_005517	*Homo sapiens* high mobility group nucleosomal binding domain 2
LMBRD1	NM_018368	*Homo sapiens* LMBR1 domain containing 1
PMEL (SILV)	NM_006928	*Homo sapiens* premelanosome protein (*Homo sapiens* silver homolog (mouse))
FRMD5	NM_032892	FERM domain containing 5
CENPA	NM_001809	*Homo sapiens* centromere protein A
CDKN2A	NM_058197	*Homo sapiens* cyclin-dependent kinase inhibitor 2A (melanoma, p16, inhibits CDK4)
RDM1	NM_001034836	*Homo sapiens* RAD52 motif 1
QPCT	NM_012413	*Homo sapiens* glutaminyl-peptide cyclotransferase
GNAQ	NM_002072	*Homo sapiens* guanine nucleotide binding protein (G protein), q polypeptide
ARHGAP32	NM_014715	*Homo sapiens* Rho GTPase activating protein 32
