# Supplementary material for: Comparison of the Transcriptional Profiles of Melanocytes from Dark and Light Skinned Individuals under Basal Conditions and Following Ultraviolet-B Irradiation
Source: PLoS One. 2015 Aug 5;10(8):e0134911. doi: 10.1371/journal.pone.0134911 (PMC4526690; doi:10.1371/journal.pone.0134911)
Supplement: S2 Table — (PDF) [file pone.0134911.s002.pdf]

S2 Table.Top 50 upregulated genes in DM vs LM 12 hours after UVB (non- coding RNAs are indicated with *) (bonferroni-adjusted p-value <0.0001).

Locus name	Accession number	Description
KPNB1	NM_002265	*Homo sapiens* karyopherin (importin) beta 1
NUP50	NM_007172	*Homo sapiens* nucleoporin 50kDa
GABPB1	NM_002041	*Homo sapiens* GA binding protein transcription factor, beta subunit 1
CACHD1	NM_020925	*Homo sapiens* cache domain containing 1
NOM1	NM_138400	*Homo sapiens* nucleolar protein with MIF4G domain 1
LRRC42	NM_029985	Leucine rich repeat containing 42
GET4	NM_015949	*Homo sapiens* golgi to ER traffic protein 4 homolog (S. cerevisiae)
C16orf55	NM_153025	*Homo sapiens* chromosome 16 open reading frame 55
MRPL15	NM_014175	*Homo sapiens* mitochondrial ribosomal protein L15
MT1X	NM_005952	*Homo sapiens* metallothionein 1X
**LOC399744*	NR_024497	
CPSF3L	NM_017871	*Homo sapiens* cleavage and polyadenylation specific factor 3-like
ZNF498	NM_145115	*Homo sapiens* zinc finger protein 498
**LOC151174*	NR_026925	
UPF3A	NM_023011	*Homo sapiens* UPF3 regulator of nonsense transcripts homolog A
SSR3	NM_007107	*Homo sapiens* signal sequence receptor, gamma
DAB1	NM_021080	*Homo sapiens* disabled homolog 1 (Drosophila)
HOOK3	NM_032410	*Homo sapiens* hook homolog 3 (Drosophila)
MFSD8	NM_152778	*Homo sapiens* major facilitator superfamily domain containing 8
CHRD	NM_003741	*Homo sapiens* chordin
**LOC100129280*	AK126613	
MAD2L2	NM_027985	MAD2 mitotic arrest deficient-like 2 (yeast)
GCN1L1	NM_006836	*Homo sapiens* GCN1 general control of amino-acid synthesis 1-like 1 (yeast)
HMX2	NM_005519	*Homo sapiens* H6 family homeobox 2
AAMP	NM_001087	*Homo sapiens* angio-associated, migratory cell protein
ANKRD30B	NM_001145029	*Homo sapiens* ankyrin repeat domain 30B
AVIL	NM_006576	*Homo sapiens* advillin
STAG2	NM_001042749	*Homo sapiens* stromal antigen 2
GBA2	NM_020944	*Homo sapiens* glucosidase, beta (bile acid) 2
MAP2K4	NM_003010	*Homo sapiens* mitogen-activated protein kinase kinase 4
SDF2	NM_006923	*Homo sapiens* stromal cell-derived factor 2
CPT1B	NM_152246	*Homo sapiens* carnitine palmitoyltransferase 1B (muscle)
SH3D21	NM_001162530	*Homo sapiens* SH3 domain containing 21
C10orf96	NM_198515	*Homo sapiens* chromosome 10 open reading frame 96
STARD3	NM_001165937	*Homo sapiens* StAR-related lipid transfer (START) domain containing 3
**LOC339807*	NR_034023	
B3GALNT2	NM_152490	*Homo sapiens* beta-1,3-N-acetylgalactosaminyltransferase 2
SRM	NM_003132	*Homo sapiens* spermidine synthase
ZNF562	NM_017656	*Homo sapiens* zinc finger protein 562
CCT8	NM_006585	*Homo sapiens* chaperonin containing TCP1, subunit 8 (theta)
MPDU1	NM_004870	*Homo sapiens* mannose-P-dolichol utilization defect 1
POLR3A	NM_007055	*Homo sapiens* polymerase (RNA) III
ENY2	NM_020189	*Homo sapiens* enhancer of yellow 2 homolog (Drosophila)
C3orf19	NM_016474	*Homo sapiens* chromosome 3 open reading frame 19
MRPS18A	NM_018135	*Homo sapiens* mitochondrial ribosomal protein S18A
DENND3	NM_014957	DENN/MADD domain containing 3
F11R	NM_016946	*Homo sapiens* F11 receptor
HERC5	NM_016323	*Homo sapiens* hect domain and RLD 5
ADM2	NM_024866	*Homo sapiens* adrenomedullin 2
ASXL2	NM_018263	*Homo sapiens* additional sex combs like 2 (Drosophila)
