# Supplementary material for: Comparison of the Transcriptional Profiles of Melanocytes from Dark and Light Skinned Individuals under Basal Conditions and Following Ultraviolet-B Irradiation
Source: PLoS One. 2015 Aug 5;10(8):e0134911. doi: 10.1371/journal.pone.0134911 (PMC4526690; doi:10.1371/journal.pone.0134911)
Supplement: S3 Table — (PDF) [file pone.0134911.s003.pdf]

S3 Table.Top 50 upregulated genes in LM vs DM 24 hours after UVB (non- coding RNAs are indicated with *) (bonferroni-adjusted p-value <0.0001).

Locus name	Accession number	Description
PRKG1	NM_006258	*Homo sapiens* protein kinase, cGMP-dependent, type I
ALPL	NM_000478	*Homo sapiens* alkaline phosphatase, liver/bone/kidney
BCAM	NM_001013257	*Homo sapiens* basal cell adhesion molecule (Lutheran blood group)
DEFB121	NM_001171832	*Homo sapiens* defensin, beta 121
**HCG9*	NR_028032	*Homo sapiens* HLA complex group 9
C11orf66	NM_145017	*Homo sapiens* chromosome 11 open reading frame 66
FLJ36848	AK094167	*Homo sapiens* cDNA FLJ36848 fis, clone ASTRO2013802.
CCDC85A	NM_001080433	*Homo sapiens* coiled-coil domain containing 85A
NR4A1	NM_002135	*Homo sapiens* nuclear receptor subfamily 4, group A, member 1
**DKFZP434K028*	NR_026882	*Homo sapiens* hypothetical LOC26070
GREM1	NM_013372	*Homo sapiens* gremlin 1
ELTD1	NM_022159	*Homo sapiens* EGF, latrophilin and seven transmembrane domain containing 1
PLCB1	NM_015192	*Homo sapiens* phospholipase C, beta 1
NPTX2	NM_002523	*Homo sapiens* neuronal pentraxin II
ASPN	NM_001193335	*Homo sapiens* asporin
COX8C	NM_182971	*Homo sapiens* cytochrome c oxidase subunit VIIIC
C12orf42	Source:HGNC Symbol;Acc:24729	chromosome 12 open reading frame 42
AMZ1	NM_133463	*Homo sapiens* archaelysin family metallopeptidase 1
**LOC441495*	NR_033773	*Homo sapiens* centromere protein V pseudogene
LHX2	NM_004789	*Homo sapiens* LIM homeobox 2
SLC22A12	NM_144585	*Homo sapiens* solute carrier family 22 (organic anion/urate transporter), member 12
ARID3C	NM_001017363	*Homo sapiens* AT rich interactive domain 3C BRIGHT-like)
DCDC1	NM_181807	*Homo sapiens* doublecortin domain containing 1
IL22RA1	NM_021258	*Homo sapiens* interleukin 22 receptor, alpha 1
**SNAR-B1*	BF570972	
SLA	NM_001045556	*Homo sapiens* Src-like-adaptor
**TPRXL*	NR_002223	*Homo sapiens* tetra-peptide repeat homeobox-like
C9orf152	NM_001012993	*Homo sapiens* chromosome 9 open reading frame 152
**XLOC_002736*		
LTBP1	NM_206943	*Homo sapiens* latent transforming growth factor beta binding protein 1
CYP2W1	NM_017781	*Homo sapiens* cytochrome P450, family 2, subfamily W, polypeptide 1
CXADR	NM_001338	*Homo sapiens* coxsackie virus and adenovirus receptor
LRRTM2	NM_015564	*Homo sapiens* leucine rich repeat transmembrane neuronal 2
GRM2	NM_000839	*Homo sapiens* glutamate receptor, metabotropic 2
PCSK1	NM_000439	*Homo sapiens* proprotein convertase subtilisin/kexin type 1
**NCRNA00242*	NR_026781	*Homo sapiens* non-protein coding RNA 242
ZNF711	NM_021998	*Homo sapiens* zinc finger protein 711
COX3	NC_012920.1	mitochondrially encoded cytochrome c oxidase III
POTEF	NM_001099771	*Homo sapiens* POTE ankyrin domain family, member F
RPL22L1	NM_001099645	*Homo sapiens* ribosomal protein L22-like 1
AGMO	NM_001004320	*Homo sapiens* alkylglycerol monooxygenase
COL4A2	NM_001846	*Homo sapiens* collagen, type IV, alpha 2
FOXD1	NM_004472	*Homo sapiens* forkhead box D1
FUT6	NM_000150	*Homo sapiens* fucosyltransferase 6 (alpha (1,3) fucosyltransferase)
MAL	NM_002371	*Homo sapiens* mal, T-cell differentiation protein
SMS	NM_004595	*Homo sapiens* spermine synthase
HSPA8	NM_153201	*Homo sapiens* heat shock 70kDa protein 8
PYGL	NM_002863	*Homo sapiens* phosphorylase, glycogen, liver
ACTN1	NM_001102	*Homo sapiens* actinin, alpha 1
VGLL3	NM_016206	*Homo sapiens* vestigial like 3 (Drosophila)
