# Supplementary material for: Comparison of the Transcriptional Profiles of Melanocytes from Dark and Light Skinned Individuals under Basal Conditions and Following Ultraviolet-B Irradiation
Source: PLoS One. 2015 Aug 5;10(8):e0134911. doi: 10.1371/journal.pone.0134911 (PMC4526690; doi:10.1371/journal.pone.0134911)
Supplement: S4 Table — (PDF) [file pone.0134911.s004.pdf]

S4 Table.Top 50 upregulated genes in DM vs LM 24 hours after UVB (non- coding RNAs are indicated with *) (bonferroni-adjusted p-value <0.0001).

Locus name	Accession number	Description
EPT1	NM_033505	*Homo sapiens* ethanolaminephosphotransferase 1 (CDP-ethanolamine-specific)
NFYC	NM_014223	*Homo sapiens* nuclear transcription factor Y, gamma
OCIAD1	NM_001079839	*Homo sapiens* OCIA domain containing 1
L3MBTL1	NM_032107	*Homo sapiens* l (3)mbt-like 1 (Drosophila)
EPB41L4A	NM_022140	*Homo sapiens* erythrocyte membrane protein band 4.1 like 4A
PTPN1	NM_002827	*Homo sapiens* protein tyrosine phosphatase, non-receptor type 1
ZAK	NM_133646	*Homo sapiens* sterile alpha motif and leucine zipper containing kinase AZK
PRADC1	NM_032319	*Homo sapiens* protease-associated domain containing 1
UTP6	NM_018428	*Homo sapiens* UTP6, small subunit (SSU) processome component, homolog (yeast)
NAA20	NM_016100	*Homo sapiens* N (alpha)-acetyltransferase 20, NatB catalytic subunit
AEBP2	NM_153207	*Homo sapiens* AE binding protein 2
PLS1	NM_002670	*Homo sapiens* plastin 1
SH3KBP1	NM_001024666	*Homo sapiens* SH3-domain kinase binding protein 1
CUL4A	NM_001008895	*Homo sapiens* cullin 4A
C19orf2	NM_003796	*Homo sapiens* chromosome 19 open reading frame 2
FDXACB1	NM_138378	*Homo sapiens* ferredoxin-fold anticodon binding domain containing 1
TRDMT1	NM_004412	*Homo sapiens* tRNA aspartic acid methyltransferase 1
ESAM	NM_138961	*Homo sapiens* endothelial cell adhesion molecule
ARSK	NM_198150	*Homo sapiens* arylsulfatase family, member K
CYP4A11	NM_000778	*Homo sapiens* cytochrome P450, family 4, subfamily A, polypeptide 11
MED23	NM_015979	*Homo sapiens* mediator complex subunit 23
KIF1A	NM_004321	*Homo sapiens* kinesin family member 1A
SLC28A1	NM_004213	*Homo sapiens* solute carrier family 28 (sodium-coupled nucleoside transporter), member 1
PHF10	NM_018288	*Homo sapiens* PHD finger protein 10
PEX26	NM_001199319	*Homo sapiens* peroxisomal biogenesis factor 26
PPY	NM_002722	*Homo sapiens* pancreatic polypeptide
FAM189A2	NM_004816	*Homo sapiens* family with sequence similarity 189, member A2
ATP2A3	NM_174953	*Homo sapiens* ATPase, Ca++ transporting, ubiquitous
HLA-DPB1	NM_002121	*Homo sapiens* major histocompatibility complex, class II, DP beta 1
ABT1	NM_013375	*Homo sapiens* activator of basal transcription 1
PTK7	NM_002821	*Homo sapiens* PTK7 protein tyrosine kinase 7
**LOC642781*	XR_112733	PREDICTED: *Homo sapiens* hypothetical LOC642781
RAP2A	NM_021033	*Homo sapiens* RAP2A, member of RAS oncogene family
ANKRD13C	NM_030816	*Homo sapiens* ankyrin repeat domain 13C
PPP6C	NM_002721	*Homo sapiens* protein phosphatase 6, catalytic subunit
FTSJ3	NM_017647	*Homo sapiens* FtsJ homolog 3 (E.coli)
RNF113A	NM_006978	*Homo sapiens* ring finger protein 113A
RTN3	NM_006054	*Homo sapiens* reticulon 3
MAPKAP1	NM_001006618	*Homo sapiens* mitogen-activated protein kinase associated protein 1
LOC643669	XM_933621	PREDICTED: *Homo sapiens* hypothetical protein LOC643669, transcript variant 1
CLSTN3	NM_014718	*Homo sapiens* calsyntenin 3
THBS2	NM_003247	*Homo sapiens* thrombospondin 2
**LOC254559*	NR_015411	*Homo sapiens* hypothetical LOC254559
PPFIBP1	NM_177444	*Homo sapiens* PTPRF interacting protein, binding protein 1 (liprin beta 1)
**ZNF284*	CR936662	*Homo sapiens* mRNA; cDNA DKFZp781F1775
SIRT6	NM_016539	*Homo sapiens* sirtuin 6
RABGAP1	NM_012197	RAB GTPase activating protein 1
C10orf58	NM_032333	*Homo sapiens* chromosome 10 open reading frame 58
FCRLB	NM_001002901	*Homo sapiens* Fc receptor-like B
